# Identification of differentially expressed genes and pathways in mice exposed to mixed field neutron/photon radiation

**DOI:** 10.1186/s12864-018-4884-6

**Published:** 2018-06-28

**Authors:** Constantinos G. Broustas, Andrew D. Harken, Guy Garty, Sally A. Amundson

**Affiliations:** 1 Center for Radiological Research, Columbia University Medical Center, 630 West 168th Street, New York, NY 10032 USA; 2 Radiological Research Accelerator Facility, Columbia University, Irvington, NY 10533 USA

**Keywords:** Mixed field neutron/photon, Gene expression, Radiation biodosimetry, Mouse blood

## Abstract

**Background:**

Radiation exposure due to the detonation of an improvised nuclear device remains a major security concern. Radiation from such a device involves a combination of photons and neutrons. Although photons will make the greater contribution to the total dose, neutrons will certainly have an impact on the severity of the exposure as they have high relative biological effectiveness.

**Results:**

We investigated the gene expression signatures in the blood of mice exposed to 3 Gy x-rays, 0.75 Gy of neutrons, or to mixed field photon/neutron with the neutron fraction contributing 5, 15%, or 25% of a total 3 Gy radiation dose. Gene ontology and pathway analysis revealed that genes involved in protein ubiquitination pathways were significantly overrepresented in all radiation doses and qualities. On the other hand, eukaryotic initiation factor 2 (EIF2) signaling pathway was identified as one of the top 10 ranked canonical pathways in neutron, but not pure x-ray, exposures. In addition, the related mTOR and regulation of EIF4/p70S6K pathways were also significantly underrepresented in the exposures with a neutron component, but not in x-ray radiation. The majority of the changed genes in these pathways belonged to the ribosome biogenesis and translation machinery and included several translation initiation factors (e.g. *Eif2ak4*, *Eif3f*), as well as 40S and 60S ribosomal subunits (e.g. *Rsp19*, *Rpl19*, *Rpl27*). Many of the differentially downregulated ribosomal genes (e.g. *RPS19*, *RPS28*) have been causally associated with human bone marrow failure syndromes and hematologic malignancies. We also observed downregulation of transfer RNA processes, in the neutron-only exposure (*p* < 0.005). Ingenuity Pathway Analysis (*p* < 0.05) of differentially expressed genes predicted significantly suppressed activity of the upstream regulators *c-Myc* and *Mycn,* transcription factors known to control ribosome biogenesis.

**Conclusions:**

We describe the gene expression profile of mouse blood following exposure to mixed field neutron/photon irradiation. We have discovered that pathways related to protein translation are significantly underrepresented in the exposures containing a neutron component. Our results highlight the significance of neutron exposures that even the smallest percentage can have profound biological effects that will affect medical management and treatment decisions in case of a radiological emergency.

**Electronic supplementary material:**

The online version of this article (10.1186/s12864-018-4884-6) contains supplementary material, which is available to authorized users.

## Background

Radiation exposure due to the detonation of an improvised nuclear device poses a growing threat to human health. In such a scenario, large populations will be exposed to various doses of radiation. A number of fatalities will occur immediately after the incident, whereas an even larger number of individuals will experience long-term effects such as higher incidence of cancer, infections and other diseases. However, many people at a distance from the directly affected area will also receive radiation that poses no immediate health risk. The goal of radiation biodosimetry is to quickly and accurately evaluate radiation dose as a means to assess radiological injury. A wide variety of radiation biodosimetry methods has been developed, ranging from cytogenetic assays [1] to the more recently proposed miRNA [2], metabolomics [3], and gene expression analyses. Unbiased transcriptomic profiling has been used to assess individual exposure to radiation. We and others have focused on peripheral blood cells for the development of transcriptomic approaches. Global gene expression in blood from mice exposed to photons (x-rays, γ-rays) has shown that gene expression changes with radiation dose, time, and dose rate [4–6]. Although radiation quality is expected to play an important role in driving gene expression, and different signaling pathways may be triggered in response to the different types of irradiation, a limited number of such studies have been performed. For example, a small number of studies have focused on comparing gene expression profiles in various tissues between photon and heavy ions or α-particles, which could be relevant to a radiological dispersal device. Depending on the cell type, similar or distinct gene expression profiles have been described [7–9].

The detonation of an improvised nuclear device could expose individuals to a mixture of photon and neutron irradiation. Although most of the radiation dose will come from photons, a small part of it will be due to neutrons; however, neutron’s potential of radiological injury will be disproportionally large, since neutrons have high relative biological effectiveness (RBE), as they are able to generate DNA damage that is irreparable or difficult to repair [10].

We previously compared the gene expression signatures in blood of mice exposed separately to either x-rays or neutrons at 1 and 7 days post-irradiation [11]. Our analysis showed differential gene expression effects of neutrons versus photons. Gene ontology analysis revealed that genes involved in nucleic acid metabolism processes were downregulated in neutron-irradiated mice, whereas genes involved in lipid metabolism were upregulated in x-ray irradiated animals. Cell cycle processes were significant among down-regulated genes at day 1, but among up-regulated genes at day 7 after exposure to either neutron or x-rays.

There have been a limited number of publications describing the effect of mixed neutron/photon field irradiation on the survival of various strains of mice and non-human primates [12–17]. In these studies the neutron component ranges from 50 to 70% of the total radiation dose, somewhat higher than the proportion of neutron dose expected in an IND. These studies have shown that exposure to neutron/photon irradiation results in increased defects in T cell populations, greater susceptibility to bacterial infections, and reduced overall survival compared with animals irradiated with pure photons.

In the present study, we analyzed transcriptomic changes over a range of mixed neutron/photon radiation doses with the neutron component contributing 5, 15, 25%, of the total dose of 3 Gy. For comparison, we also included mice exposed to 3 Gy of pure x-rays or 0.75 Gy neutron radiations (equivalent to the neutron component in the 83% neutron exposure). To mimic the radiation conditions of an IND detonation, we used the broad-spectrum neutron irradiator at the Columbia University Radiological Research Accelerator Facility (RARAF) that is capable of generating neutrons with an energy spectrum mirroring that produced at 1–1.5 km from the epicenter of the Hiroshima blast [18, 19]. A small percentage of photons (~ 17%) contributes to total radiation dose generated by the neutron source [19].

To our knowledge this is the first description of the gene expression patterns in mouse peripheral blood after a mixed-field radiation exposure. Our ultimate goal is to investigate whether gene expression signatures can accurately identify the neutron component in a mixed field exposure.

## Methods

### Animals and irradiation

All animal experiments were conducted in accordance with applicable federal and state guidelines and were approved by the Columbia University Institutional Animal Care and Use Committee (IACUC) (approval number AC-AAAG4356). Male C57BL/6 mice were received from Charles River Laboratories (Frederick, MD) and quarantined for 2 weeks before irradiation at 8–10 weeks of age. Six mice were used for each treatment and sacrificed 7 days after treatment.

Mice were either sham-irradiated or exposed to x-ray, neutron or mixed field neutron/x-ray radiation as detailed in Table 1. The neutron source delivers a small percentage of photons, as well. Therefore the maximum achievable dose of neutrons is approximately 83%. Neutron irradiations were performed using the accelerator-based IND-spectrum neutron source [19] at the Radiological Research Accelerator Facility (RARAF). The characteristics of the neutron source and the mouse exposure protocol have been detailed previously [11].

**Table 1:** Protocol of mixed field neutron/x-ray exposure

Neutron Dose, Gy	Photon dose	x-ray dose	Total dose	Neutron Fraction
0	0	3.00	3.00	0%
0.15	0.03	2.82	3.00	5%
0.45	0.08	2.47	3.00	15%
0.75	0.13	2.12	3.00	25%
0.75	0.13	0	0.75	83%

For x-ray irradiation, mice were exposed to various doses of x-rays from a Westinghouse Coronado orthovoltage x-ray machine operating at 250 kVp and 15 mA with a 0.5 mm Cu + 1 mm Al filter as described previously [11]. After dose administration, mice were housed in micro-isolator cages until the time of sacrifice by carbon dioxide asphyxiation followed by cervical dislocation.

### Blood collection and RNA isolation

Blood was collected by cardiac puncture at the time of euthanasia at day 7 post-irradiation. Each sample (~ 0.4 ml blood) was added to a 15 ml centrifuge tube that contained 1.6 ml of PAXgene Blood RNA stabilization and lysis solution (PreAnalytix GmBH, catalog # 762165) and mixed thoroughly, while a small amount of blood was added to tubes containing EDTA as anti-coagulant for blood count using a Genesis hematology system (Oxford Science). After collection was complete, blood was mixed gently but thoroughly and the tubes were incubated at -20 °C for 2 days. Blood samples were allowed to reach room temperature for 2 h before proceeding to RNA isolation. RNA was purified following the PAXgene RNA kit recommendations with on-column DNase I treatment. Excess globin RNA was reduced using the Ambion GLOBINclear-mouse/rat kit (Thermofisher). RNA yields were quantified using the NanoDrop ND1000 spectrophotometer (Thermofisher) and RNA quality was checked by the 2100 Bioanalyzer (Agilent). High quality RNA with an RNA integrity number of at least 7.0 was used for microarray hybridization.

### Microarray hybridization and data analysis

Cyanine-3 labeled cRNA was prepared using the One-Color Low Input Quick Amp Labeling kit (Agilent). Dye incorporation and cRNA yield were measured with a NanoDrop ND1000 spectrophotometer (Thermofisher). Labeled cRNA was fragmented and hybridized to Agilent Mouse Gene Expression 4x44K v2 Microarray Kit (G4846A). Slides were scanned with the Agilent DNA microarray scanner (G2505B) and the images were analyzed with Feature Extraction software (Agilent) using default parameters for background correction and flagging non-uniform features.

Background-corrected hybridization intensities were imported into BRB-ArrayTools, version 4.5.0, log2-transformed and median normalized. Non-uniform outliers or features not significantly above background intensity in 25% or more of the hybridizations were filtered out. In addition, a minimum 1.5-fold change in at least 20% of the hybridizations was set as a requirement. Furthermore, probes were averaged to one probe per gene and duplicate features were reduced by selecting the one with maximum signal intensity. A final set of 14,243 genes that passed the filtering criteria was used in subsequent analyses. The microarray data is available through the Gene Expression Omnibus with accession number GSE113509.

Class comparison was conducted in BRB-ArrayTools to identify genes differentially expressed (*p* < 0.001) between radiation exposed samples and time-matched unirradiated controls using a random variance t-test [20]. Genes with *p*-values less than 0.001 were considered statistically significant. The false discovery rate (FDR) was estimated for each gene by the method of Benjamini and Hochberg [21], to control for false positives. All genes used in this analysis had a FDR of less than 0.05.

Hierarchical clustering of microarray gene expression data was performed with the Dynamic Heatmap Viewer of the BRB-ArrayTools software using a one minus correlation metric and average linkage.

### Gene ontology analysis

Lists of genes that were either significantly overexpressed or underexpressed compared with controls were analyzed using the Ingenuity Pathway Analysis (IPA) core pathway and upstream regulator analysis (Qiagen Ingenuity Systems). Furthermore, the list of differentially expressed genes was also analyzed using Protein ANalysis THrough Evolutionary Relationships (PANTHER), version 11 [22]. Benjamini corrected *p* values of < 0.05 were considered significant.

### Quantitative RT-PCR

cDNA was prepared from total globin-cleared RNA using the High-Capacity cDNA Archive kit (Thermofisher). Quantitative real-time PCR (qRT-PCR) was performed for six genes using pre-designed validated Taqman assays (Thermofisher). These genes were *Crip2* (Mm00517877_m1), *Chst3* (Mm00489736_m1), *Add3* (Mm00507934_m1), Eif3f (Mm00517953_m1), *Rpl26* (Mm02343715_g1), *Rpl27* (Mm01245874_g1), *Rps17* (Mm01314921_g1), *Rps19* (Mm01611010_g1), and *c-Myc* (Mm00487804_m1). A β-actin assay (Mm00607939_s1) was also performed alongside as control. The gene expression validation experiments were conducted with 20 ng cDNA using Universal PCR Master Mix (Thermofisher) in an ABI 7900 Real Time PCR system. After an initial activation at 50 °C for 2 min and 95 °C for 10 min, the PCR reaction was performed by 40 cycles of 95 °C for 15 s and 60 °C for 60 s. Relative fold-induction was calculated by the 2^-ΔΔCT^ method [23], using SDS version 2.3 (Thermofisher). Data were normalized to β-actin gene expression levels.

## Results

### Microarray experiments

We recently described the gene expression profiles of mouse blood cells following either x-ray or neutron irradiation and analyzed them 1 and 7 days post-irradiation. In that report, we found that neutron radiation results in mainly distinct gene expression patterns compared with x-ray irradiation [11].

In the current study, we investigated whether we can identify the neutron component after a mixed field x-ray/neutron exposure, by analyzing gene expression signatures. Thus, mice were either sham-irradiated or exposed to 3 Gy of mixed neutron/x-ray exposures that contained 5, 15, and 25% of neutron component (Table 1). For comparison, mice were also exposed to 3 Gy x-rays or 0.75 Gy of neutron irradiation (equivalent to the neutron component in the 83% neutron exposure). All animals remained in apparent good health, with no adverse events noted during the course of the study.

**Fig. 1:**
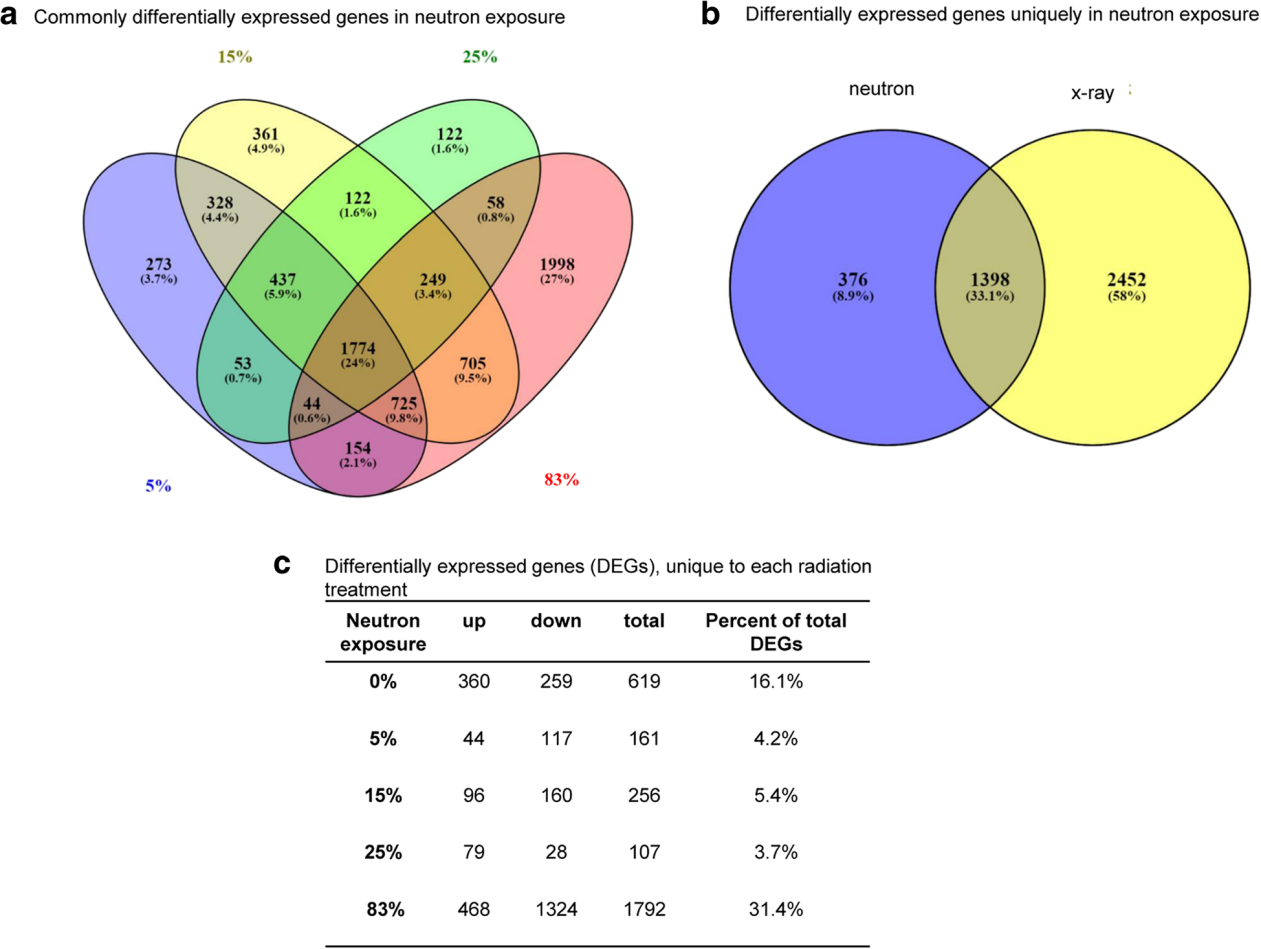
Differentially expressed genes. **a** Venn diagram showing overlap patterns of genes that are differentially expressed in response to 0.75 Gy neutron (83%), or 3 Gy mixed-field neutron/x-ray (5, 15, 25%). **b** Venn diagram comparing differentially expressed genes in x-rays (x-ray) versus neutron or neutron/x-ray (neutron) exposures. **c** Table showing absolute number and percent of differentially expressed genes (DEGs) unique to each treatment

Global gene expression was measured in the blood of mice sacrificed 7 days post-irradiation using Agilent Whole Mouse Genome Microarrays. Class comparison using BRB-ArrayTools identified a total of 3850, 3788, 4701, 2859, and 5707 genes differentially expressed (*p* < 0.001, false discovery rate (FDR) < 5%) between unirradiated controls and x-ray, 5, 15, 25% mixed field x-ray/neutron, and 83% neutron exposed samples, respectively (Table 2 and Additional file 1). Following exposures, the majority of the genes were down-regulated. In our previous study [11], we had exposed mice to x-ray or neutron irradiation and had identified a number of genes, including *Fzr1*, *Ube2c*, and *Ccna2* that were upregulated at day 7 post-irradiation. We confirmed in the present study that these genes were indeed among the list of upregulated genes in the 3 Gy x-ray and 0.75 Gy neutron exposures. Among the exposures that contained a neutron component, 1774 significantly differentially expressed genes were identified (Fig. 1a). Of these genes 376 were uniquely differentially expressed only in neutron, but not in x-rays (Fig. 1b). Unique genes for each particular exposure were also identified (Fig. 1c). Not surprisingly, few genes were unique to the 5, 15, and 25% exposures. On the other hand, approximately one third of all differentially expressed genes in the 83% neutron exposure were unique to that exposure. The overall number of genes responding to the 25% neutron/x-ray exposure was significantly lower than other percentages, reflecting mainly the lower number of downregulated genes, although the number of the upregulated genes was comparable. A dose-response effect was not evident in the majority of differentially exposed genes in the neutron exposures. Instead, differentially upregulated genes showed a progressive increase in the fold-change that peaked at 15% neutron/photon. Likewise, downregulated genes showed the maximum fold-change again at 15% neutron/photon.

**Table 2:** Differentially expressed genes

neutron	up	down	total	%up	%down
0%	1373	2477	3850	35.7	64.3
5%	829	2959	3788	21.9	78.1
15%	1417	3284	4701	30.1	69.9
25%	1052	1807	2859	36.8	63.2
83%	1390	4317	5707	24.4	75.6

Significantly differentially expressed genes in mouse blood after x-ray, neutron, or mixed field x-ray/neutron treatment relative to unirradiated controls (p < 0.001). Percent of upregulated (up) and downregulated (down) genes are shown

### Gene ontology analysis

**Fig. 2:**
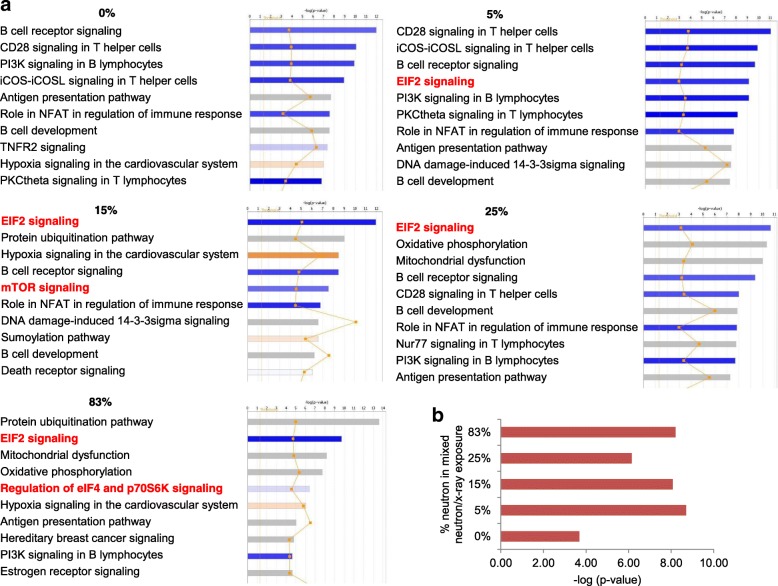
**a** Ingenuity Pathway Analysis (IPA) top 10 canonical pathways for each treatment (*p* < 0.001). The most statistically significant canonical pathways identified in the neutron exposure are listed according to their *p* value (−log; orange line). *Blue bars*: negative z-score; *orange bars*: positive z-score; *gray bars*: no activity pattern available. The orange squares represent the ratio of the number of differentially expressed genes found in each pathway over the total number of genes in that pathway. **b** IPA on EIF2-related differentially expressed genes plotted according their *p*-value (−log)

We used Ingenuity Pathway Analysis (IPA) to identify the most significantly enriched molecular functions. Biological processes and pathways over-represented among differentially expressed genes with a Benjamini-corrected *p*-value of less than 0.05 were considered significant. The top ten canonical pathways based on the significance of the enrichment (p-value) are shown in Fig. 2. Canonical pathways pertaining to B- and T-cell immune response were uniformly underrepresented in all radiation exposures (Fig. 2a). In contrast, the “EIF2 signaling” pathway was significantly underrepresented only in the exposures that included a neutron component (Fig. 2a and Additional file 2). If the additional criterion of fold-change ±2 was applied to the IPA analysis, the “EIF2 signaling” pathway was among the significantly enriched pathways in the mixed-field and 83% neutron exposure, but interestingly not in the pure x-ray exposure (Additional file 3 Figure S1), thus underlining the robustness of the study. In addition, the related “mTOR signaling” and “regulation of eIF4/p70S6K1 signaling” pathways were also significantly underrepresented in the 15 and 83% exposures. A summary of –log (*p*-value) is shown (Fig. 2b).

The PANTHER gene ontology tool was used as an additional method for evaluating functional significance of the radiation-induced gene profiles. Analyzing the results (*p* < 0.005; Additional file 4) confirmed that among top pathways significantly underrepresented in the neutron exposures were those related to EIF2 signaling (Table 3).

**Table 3:** mRNA Translation-related biological processes in response to radiation as identified by PANTHER analysis (*p* < 0.005)

Term	0%	5%	15%	25%	83%
eukaryotic initiation factor 4E binding (GO:0008190)	3.09E-02		3.79E-02		6.40E-03
translation initiation factor binding (GO:0031369)		2.63E-03		1.86E-03	1.02E-05
eukaryotic initiation factor eIF2 binding (GO:0071074)		1.60E-02			3.67E-02
translation initiation factor activity (GO:0003743)		2.99E-02	6.78E-04	1.75E-02	2.01E-06
translation elongation factor activity (GO:0003746)					2.06E-02
translation termination factor activity (GO:0008079)					3.92E-02
translation release factor activity (GO:0003747)					3.92E-02

Significantly enriched biological processes among genes downregulated in response to radiation. Benjamini-corrected p values are shown for each radiation exposure

**Fig. 3:**
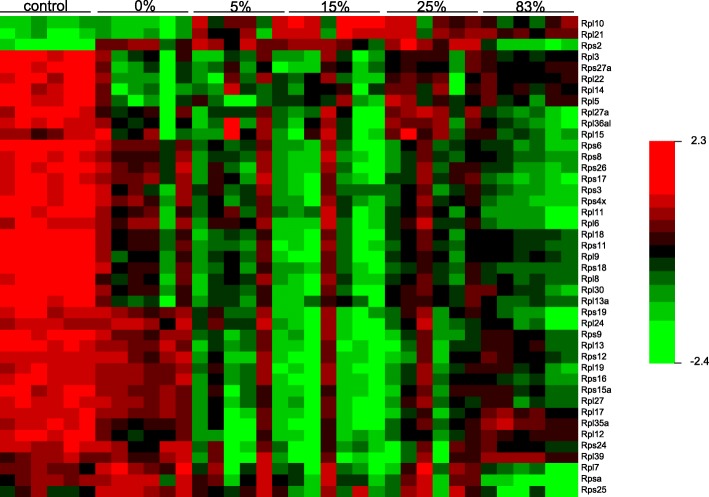
Heatmap illustrating relative gene expression of ribosomal genes among the differentially expressed genes (p < 0.001) at various radiation exposures. *Red* indicates high expression; *green* indicates low expression, as indicated in the color key. Each row represents one gene and each column represents an individual mouse

Examining the list of differentially expressed genes in the EIF2 signaling pathway (Additional file 5) we noticed that several genes coding for eukaryotic initiation factors (eIFs) were mostly downregulated after irradiation. Interestingly, some eIFs (*Eif2b4*, *Eif2s3x*, and *Eif3k*) were differentially expressed after x-ray irradiation, whereas others (*Eif2ak4*, *Eif3f*, *Eif3l*) were differentially expressed after radiation that contained a neutron component (Additional file 5). Furthermore, this pathway included a large number of ribosomal protein (*RP*)-encoding genes that were downregulated in all radiation types. However, a group of 15 *RP* genes (~ 50% of all differentially regulated RP genes in this pathway) were specifically downregulated in the neutron component exposures, but not x-rays. A heatmap was constructed to visualize this observation (Fig. 3). *Rps25* was the only ribosomal gene that was differentially upregulated by x-rays, but not neutron exposure. The *RP* gene downregulation was evident even at 5% neutron and the fold-change did not vary appreciably with increasing percentage of neutron contribution. Loss-of-function, by deletion or mutation, of genes that code for ribosomal proteins are known as ribosomopathies and are causally associated with various human diseases, most prominent of which is the Diamond-Blackfan anemia. We queried the list of the neutron downregulated *RP* genes and discovered that many of these genes have been associated with the Diamond-Blackfan syndrome (Table 4).

**Table 4:** Loss-of-function ribosomal protein (RP) genes related to Diamond-Blackfan anemia and *RP* genes downregulated (DN) after neutron exposure

Diamond-Blackfan anemia		
RP gene^(1)^	X-rays	Neutrons
*RPS19*		DN
*RPL5*	DN	DN
*RPS10*		
*RPL11*	DN	DN
*RPS26*		DN
*RPL35A*		DN
*RPS24*		DN^(2)^
*RPS17*		DN
*RPS7*		
*RPL26*		DN
*RPL27*		DN
*RPL15*		
*RPS28*		DN
*RPL29*		DN
*RPL31*		
*RPS15A*		DN
*RPL35*		
*RPL18*	DN	DN
*RPS26*		
*RPS29*		
*RPS27*		

^(1)^References [32, 55]

^(2)^Only in 15 and 83% neutron exposures

Further analysis revealed that, in addition to the EIF2 pathway, biological processes related to tRNA were also significantly underrepresented in the neutron exposures, mainly in the 83% neutron exposure (Table 5). The list of tRNA-related biological processes included a number of aminoacyl-tRNA ligases (valine, threonine, arginine), enzymes responsible for charging amino acids to cognate tRNA molecules, as well as tRNA methyltranferases, essential enzymes for tRNA maturation. tRNA binding process (GO:0000049) showed progressively lower *p*-values with increasing contribution of neutrons, while this process did not attain significance after pure x-ray irradiation. Similarly, “translation factor activity, RNA binding” (GO:0008135), as well as “catalytic activity, acting on a tRNA” (GO:0140101) processes showed the same lower p-value with higher neutron percentage. An exception to this trend was the 25% exposure.

**Table 5:** tRNA-related biological processes in response to radiation as identified by PANTHER analysis (p < 0.005)

Term	0%	5%	15%	25%	83%
ligase activity, forming aminoacyl-tRNA and related compounds (GO:0016876)	3.14E-02				2.84E-05
aminoacyl-tRNA ligase activity (GO:0004812)	3.14E-02				2.84E-05
translation factor activity, RNA binding (GO:0008135)		1.62E-02	8.08E-04		8.91E-08
tRNA binding (GO:0000049)		2.99E-02	9.98E-03	6.82E-03	1.99E-07
catalytic activity, acting on a tRNA (GO:0140101)			6.71E-04	4.83E-02	4.88E-10
tRNA (guanine-N1-)-methyltransferase activity (GO:0009019)			1.88E-02		3.67E-02
tRNA-specific ribonuclease activity (GO:0004549)			2.56E-02		9.52E-03
tRNA (guanine) methyltransferase activity (GO:0016423)			3.64E-02		2.87E-03
aminoacyl-tRNA editing activity (GO:0002161)				2.62E-02	1.77E-03
valine-tRNA ligase activity (GO:0004832)					1.76E-02
threonine-tRNA ligase activity (GO:0004829)					1.76E-02
glutaminyl-tRNA synthase (glutamine-hydrolyzing) activity (GO:0050567)					1.76E-02
tRNA methyltransferase activity (GO:0008175)					3.33E-02
arginine-tRNA ligase activity (GO:0004814)					3.67E-02
tRNA dihydrouridine synthase activity (GO:0017150)					3.67E-02

Significantly enriched biological processes among genes downregulated in response to radiation. Benjamini-corrected p values are shown

Finally, PANTHER analysis revealed that processes related to protein ubiquitination were highly significant among upregulated genes in all radiation doses and qualities (Additional file 6 Table S1).

**Fig. 4:**
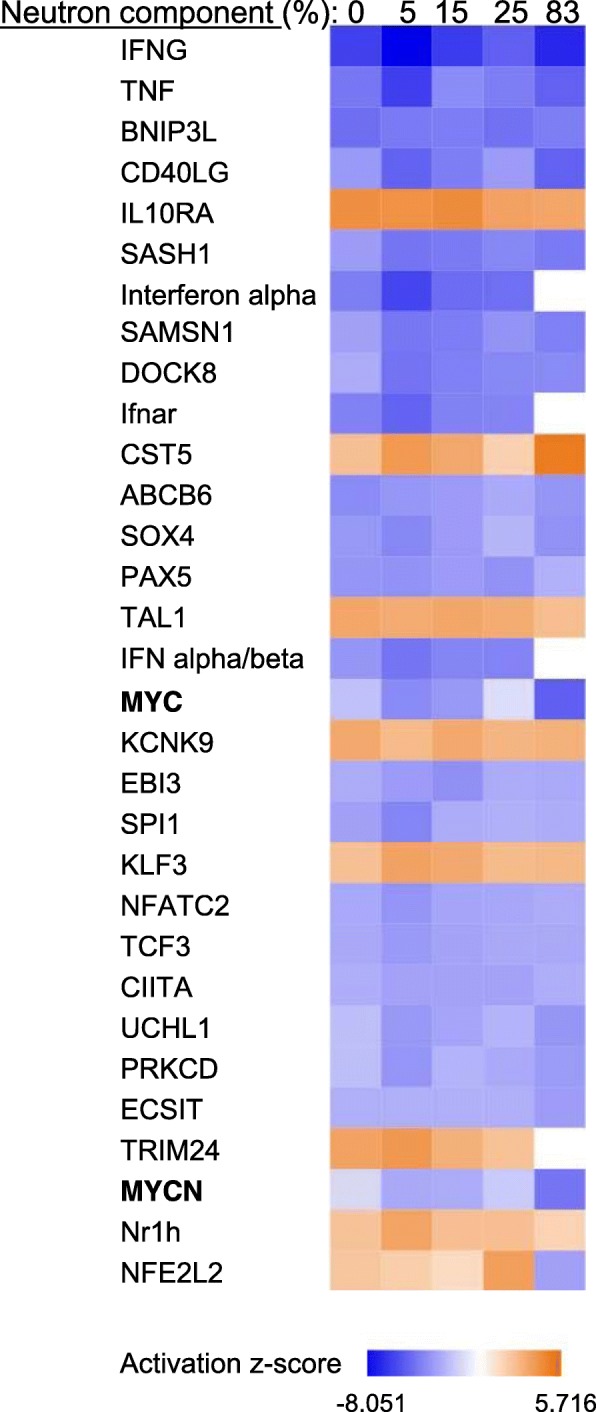
Potential upstream regulators of differentially expressed genes by neutron component using Ingenuity pathway upstream regulator. Top predicted upstream regulators (|z| > 2) are shown. *Blue* color represents negative z-scores; *orange* color represents positive z-scores as indicated in the color key. *c-Myc* and *Mycn* transcription factors are in bold

We also searched for upstream regulators of differentially regulated genes by using the upstream regulator analysis tool in IPA. This feature connects the upstream regulators with downstream functions to generate regulator effects hypotheses with predicted activation of upstream transcription regulators. We used the default z-score > 2 or z-score < − 2 as the significance cutoff for our analysis. The top candidate regulators are shown in Fig. 4 and Additional file 7. Most of the predicted upstream regulators showed similar z-scores across radiation treatments. However, *Cst5* (Cystatin D) showed a progressively larger (positive) z-score with the percentage of the neutron component. Exception was the 25% neutron/x-ray exposure that z-score did not attain statistical significance. A second candidate, *Trim24* (tripartite motif-containing 24; *Tif1*) was a positive regulator of x-rays and x-ray/lower percentage of neutron combination, but not 83% neutrons. In contrast, type I Interferon family genes, *Ifnα*, *Ifnαβ* and *Ifnar* (interferon (alpha and beta) receptor 1), important regulators of innate antiviral and antibacterial immunity [24], were predicted to be negative regulators in the exposures that contained pure photons or low percentage (5, 15%) neutrons, but not 83% neutrons. Finally, we searched for upstream regulators that function as transcription factors and identified *c-Myc* and *Mycn* as two negative regulators that displayed progressively more negative z-scores with increasing percentage of neutron component. The z-scores for *c-Myc* were − 2.0 and − 5.1 for pure x-rays and 83% neutrons, respectively, whereas for *Mycn* were − 1.3 (x-rays; not significant) and − 4.4 (83% neutrons). Again 25% neutron exposure did not predict *c-Myc* or *Mycn* as upstream regulators. The *c-Myc* gene itself was found downregulated in 5, 15, and 83% neutron exposure (*p* < 0.005) in our microarray data.

### Validation of microarray data

**Fig. 5:**
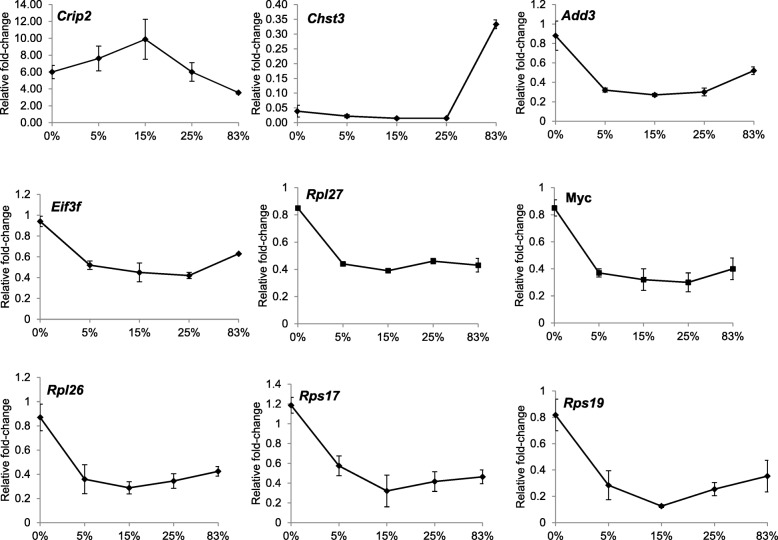
Gene expression measured by qRT-PCR. Expression of nine genes (*Crip2*, *Chst3*, *Add3*, *Eif3f*, *Rpl26*, *Rpl27*, *Rps17*, *Rps19*, and c*-Myc*) shown by microarray analysis to be differentially expressed relative to controls and normalized to *Actb* expression. Data represent the mean ± S.E.M. (*n* = 3)

We confirmed the expression pattern of some of the differentially expressed genes derived from the DNA microarray experiment, by quantitative real-time PCR (Fig. 5). We chose genes that changed in all radiation exposures (*Crip2*, *Chst3*) or were specific to neutron radiation (*Add3*, *Eif3f*, *Rpl26*, *Rpl27*, *Rps17*, *Rps19*, *c-Myc*). Analysis of gene expression of *Crip2*, *Chst3*, *Add3*, *Eif3f*, *Rpl26*, *Rpl27*, *Rps17*, *Rps19*, and *c-Myc* by qRT-PCR confirmed that these genes are regulated by irradiation. As mentioned above, differentially expressed genes showed a progressive increase in the fold-change that peaked at 15% neutron/photon. Thus, we validated *Crip2*, *Add3*, and *Chst3* by qPCR as representatives of upregulated (*Crip2*) and downregulated (*Add3*, *Chst3*) genes to demonstrate that pattern. The rest of the qPCR-validated genes belong to the “EIF2 signaling” canonical pathway. The fold-change of these genes in response to radiation was in good agreement with the fold-change calculated by the microarray experiment (Additional file 8 Figure S2). The only exception was the expression of *c-Myc* in the 25% neutron samples that appeared to be downregulated by qPCR analysis, whereas DNA microarray analysis showed no significant downregulation.

## Discussion

Previously, we described the transcriptomic profile in blood from mice exposed separately to neutron or x-ray radiation and showed that neutron radiation generates a gene expression profile that is mostly distinct from that of x-ray radiation [11]. These results suggested that differences in gene signatures between x-ray and neutron exposures could potentially be exploited for the purpose of radiation biodosimetry. However, in a real-life situation, the detonation of an IND is expected to produce a mixture of x-rays and neutrons. Although the fraction of neutrons in such a situation is not expected to exceed 25–30% [25], its biological and medical impact is predicted to be substantial due to the fact that neutron radiation has high relative biological effectiveness. Therefore, in this study, we examined the gene expression profile in mouse blood exposed to mixed field neutron/x-ray radiation. Gene expression profiles in mice exposed to 3 Gy x-rays or 0.75 Gy neutrons were similar to our previous study [11]. Genes, including *Ccna2*, *Ube2c*, and *Fzr1* that had previously been found to be upregulated at day 7 post-irradiation were also upregulated in the current study, thus confirming the reproducibility of our results. Likewise, In agreement with our current results, biological processes related to protein translation and DNA/RNA metabolism were also identified in our previous study following 83% neutron, but not pure x-ray exposure [11]. In particular, “Translation” (GO:0006412; *p* value 4.55E-11) and related pathways (GO:0006418; *p* = 0.026 and GO:0051084; *p* = 0.022) were significantly enriched in 83% neutrons but not in x-rays at day 7 post-irradiation. Furthermore, DNA/RNA metabolism processes, including tRNA processes, were identified predominantly in the 83% neutron exposures as well. For example, GO:0006399 (tRNA metabolism) was found significantly enriched among downregulated genes exposed to neutrons in both the previous (*p* = 1.37E-06), as well as the current study (*p* = 1.43E-08).

The overall number of differentially expressed genes in the mixed-field exposure was increasing with the percentage of the neutron component (Table 2). In contrast, the number of genes responding to the 25% neutron/x-ray exposure was significantly lower than the other percentages, possibly reflecting increased cell death. Indeed, in response to radiation, the number of white blood cell count is reduced considerably more in the 25% neutron/photon exposure than in the other exposures. However, to conclusively determine whether the 25% neutron/photon exposure causes the most severe effect on cell and organismal survival compared with the other exposures, future studies should focus on long term time-course survival experiments.

Mice exposed to mixed field irradiation with neutrons contributing 5, 15%, or 25% of the total exposure dose, as well as mice exposed to 83% neutrons (the highest achievable percent generated by our neutron source) identified 376 uniquely differentially expressed genes that were not shared with mice exposed to pure x-rays. However, the fold-change of these neutron-specific genes was not dependent on the percent of the neutron component.

The spectrum of biological functions and pathways within our differentially expressed genes was analyzed by using both IPA and PANTHER gene ontology analysis tools. PANTHER analysis showed that ubiquitin metabolism-related biological processes are commonly overrepresented among upregulated genes in all radiation exposures. In addition, we identified pathways related to amino acid and fatty acid oxidation to be among the significantly underrepresented pathways in the 83% neutron exposure. This finding is in agreement with recently published metabolomics data that compared x-rays to neutron radiation and found that neutrons induced a severe metabolic dysregulation, with perturbations predominantly in amino acid metabolism and fatty acid β-oxidation [26].

IPA (*p* < 0.001) and PANTHER (*p* < 0.005) analyses revealed that neutron exposure significantly suppressed processes involved in the protein translation machinery. “EIF2 signaling”, and the related pathways “mTOR signaling” and “regulation of eIF4 and p70S6K signaling” were among the top 10 significantly underrepresented canonical pathways in exposures that contained a neutron component. These processes are known to control protein synthesis by regulating mRNA translation, ribosome biogenesis, and tRNA loading [27]. mRNA translation is divided into four phases: initiation, elongation, termination, and recycling [28]. Initiation, the rate-limiting step of translation, is regulated by signaling pathways such as mTOR and p70S6K that converge on the control of eukaryotic initiation factors. Reduced translation driven by impaired ternary complex formation (eIF2, GTP, Methionine-tRNA) is encountered in conditions of specific cellular stress, that is, viral infection, amino acid deprivation, and unfolded protein response. Examining the list of differentially expressed genes in this category, we noted that most of the genes were eukaryotic initiation factors, whereas elongation or termination factors were not particularly enriched.

The most prominent distinguishing feature between x-ray and neutron exposures was the downregulation of a large number of genes that code for ribosomal proteins, including a majority of those encoding both the large (*Rpl*) and small (*Rps*) subunits, in the neutron exposures. Interestingly, a group of 15 *RP* genes was first detected downregulated at the 5% exposure, which was the lowest percentage in the mixed field exposure. Ribosomal proteins have traditionally been considered as essential but passive participants of protein synthesis. However, changes in *RP* transcript levels have been observed among different cell and tissue types [29, 30]. Furthermore, loss-of-function mutations of several ribosomal proteins lead to inherited diseases and increased tumor formation, as well as to a number of specific human disorders called ribosomopathies. Ribosomopathies are characterized by defective ribosomal function or production and include the Shwachman-Diamond syndrome, chromosome 5q-deletion syndrome, and the Diamond-Blackfan syndrome [31, 32]. These diseases initially present clinical features that can be attributed to cell proliferation defects and, depending on the affected tissue, include bone marrow failure, craniofacial malformations, bacterial infections, and mental and motor defects [31]. Intriguingly, some of these syndromes have also been associated with higher incidence of cancer later in life. In particular, Diamond-Blackfan anemia (DBA) is an autosomal dominant disorder with symptoms that include macrocytic anemia, growth retardation, craniofacial and thumb malformations, anomalies of the heart and genitourinary system, and a predisposition to develop AML (acute myeloid leukemia), as well as solid tumors [31, 33]. DBA is associated in many cases with loss-of-function of *RP* genes (Table 4). The most frequently mutated gene in DBA, which represents 25% of the cases, is that encoding *RPS19* [33]. A mouse model for DBA that contains a loss-of-function point mutation in the *Rps19* gene has been demonstrated to have a dominant negative effect [34] and, importantly, mice with *Rps19* deficiency develop bone marrow failure and symptoms like patients with Diamond-Blackfan anemia [35]. Interestingly, *Rps19* is one of the *RP* genes downregulated in the neutron or mixed neutron/photon, but not x-ray, exposed mice. Another gene downregulated specifically in irradiation exposures that contain a neutron fraction is *Rps28*. Human *RPS28* is mutated in DBA [36] and a decrease in translation of *Rps28* mRNA blocks pre-18S ribosomal RNA processing, resulting in a reduction in the number of 40S ribosomal subunits [37]. *Rpl19,* a gene that is also preferentially downregulated after mixed field irradiation, is an evolutionarily conserved gene. *RPL19* knockdown reduces growth of human prostate cancer cells both in vitro and as xenografts [38]. Furthermore, RPL19 has been proposed to be a sensitive predictor of prostate cancer progression [38, 39]. Among the neutron-regulated *RP* genes, *Rpsa* gene is the only that is differentially downregulated (~ 3-fold) in 83% neutron, but not lower percentages neutron or pure x-ray exposures. *Rpsa* mutation causes severe bacterial infections [40] and the protein can also act as a nonintegrin cell-surface receptor with high affinity for laminin with a key role in tumor invasion and metastasis [41]. Finally, *Rps25* was the only ribosomal gene that was differentially expressed by x-rays but not neutron exposure. *Rps25* expression is strongly suppressed in rat liver regeneration after partial hepatectomy, suggesting that it may have a role in liver growth [42].

Gene expression profiles of bone marrow mononuclear cells from patients with Shwachman-Diamond syndrome revealed significant downregulation of RP genes, including *RPS9*, *RPS20*, *RPL6*, *RPL15*, *RPL23*, and *RPL29*, or upregulation of RPS27 [43]. However, none of these genes are differentially expressed in response to radiation in our study. Likewise, *Rps14* loss-of-function that causes the 5q syndrome [43] was not among the differentially expressed genes.

Further analysis identified enrichment in tRNA-related biological processes that included a number of aminoacyl-tRNA ligase activities (valine, threonine, arginine tRNA ligases). Aminoacyl-tRNA ligases are responsible for charging amino acids to cognate tRNA molecules, which is the essential first step of protein translation. Mutations in genes encoding aminoacyl-tRNA ligase enzymes have been implicated in a broad array of human inherited diseases. The vast majority of these mutations show loss-of-function effects and impair protein translation [44].

In addition, tRNA methyltransferase activities were underrepresented in the 83% neutron exposure, as well. tRNAs molecules undergo extensive post-transcriptional processing to generate the mature functional tRNA species that are essential for translation in all organisms. Among these modifications, methylation reactions are the most abundant. RNA methyltransferases are a diverse group of post-transcriptional RNA modification enzymes responsible for the transfer of a methyl group from a methyl donor, most commonly S adenosylmethionine, to any of several different locations on a target RNA nucleotide. Mutations in tRNA methyltransferases have been increasingly associated with human disease [45].

We also inferred the key regulators responsible for transcriptional changes in neutron versus x-ray exposures. Among the most highly represented upstream regulators were *c-Myc* and *Mycn*. The c-Myc family of transcription factors includes three highly conserved genes, *c-Myc*, *Mycn*, and *Mycl1*. c-Myc has an evolutionarily conserved role in the control of cell size and protein synthesis [46] and regulates the transcription of a multitude of genes, including those involved in proliferation, growth, differentiation, and ribosome biogenesis [47]. Unrestrained increases in global protein synthesis can lead to tumorigenesis [48]. An examination of the differentially expressed genes revealed that *c-Myc* itself was downregulated in the 83% (*p* < 0.001), 15% (*p* < 0.005), and 5% (p < 0.005) neutron or neutron/x-ray exposures, but not in 25% or pure x-ray exposures, even when examined at the lower *p*-value. In contrast, qPCR analysis revealed that *c-Myc* is downregulated in all neutron-exposed samples. These observations reveal that *c-Myc* is significantly affected by neutron irradiation. Future genome wide analysis should aid in elucidating the mechanistic connection between *c-Myc* levels and high-LET (linear energy transfer) irradiation.

In this report we present for the first time the gene expression profiling in mouse blood exposed to mixed-filed neutron/x-ray irradiation. We have identified gene expression changes that suggest neutron exposure has a marked effect on ribosome biogenesis and mRNA translation. Furthermore, we show that a number of *RP* genes frequently defective in Blackfan-Diamond syndrome anemia are downregulated in response to neutron or mixed field irradiation, but not pure x-rays. Gene ontology and pathway analysis revealed that protein ubiquitylation pathways are enriched among upregulated genes in all radiation exposures, whereas EIF2 signaling and related pathways are enriched predominantly after neutron or mixed field irradiation. The finding that genes involved in protein synthesis are downregulated may reflect the fact that during stress, cells reduce the rate of protein synthesis, an energy-demanding process, so that they may better cope with the damage. As neutrons are known to cause greater damage in cells than a comparable dose of photons, it is, perhaps, not surprising that we find processes involved in mRNA translation to be underrepresented. We hypothesize that the downregulation of *RP* genes specifically in the neutron exposures reflects the detrimental effect of neutron exposure to bone marrow. In line with this interpretation, Cary et al. [12] have shown a decline in bone marrow cellularity 7 days after exposure to mixed field radiation.

Furthermore, our results provide insight into the potential usefulness of neutron radiation in the treatment of cancer. Accumulating evidence suggests a regulatory function for ribosomes in gene expression [49]. Dysregulation of protein synthesis by altering ribosome function impairs hematopoietic stem cell function [50] and mediates cancer or chemotherapeutic cancer resistance. Similar to the data presented here it was recently shown that some chemotherapeutic agents, exemplified by oxaliplatin, act as anti-cancer drugs not by inducing the DNA damage response pathway, but rather by inducing ribosome biogenesis stress [51]. In support of this finding, a proteomic profiling revealed that numerous ribosomal proteins are dysregulated in response to oxaliplatin treatment in T-lymphoblastic leukemia-derived cancer cells [52].

In the future, we plan studies to determine the impact of neutron and mixed field exposures on animal health and survival in comparison with animals exposed to photons. Furthermore, we intend to perform mechanistic studies to assess the genetic and phenotypic consequences of RP gene downregulation after neutron irradiation. We anticipate that the preferential downregulation of certain RP genes after irradiation containing a neutron component will change the balance in ribosomal protein composition and this may have an impact on the selectivity of translating subsets of mRNAs involved in specific biological processes as has been observed before [53]. Gene expression profiling using polysome-bound mRNA followed by microarray analysis can aid in identifying mRNA transcripts differentially expressed in mice specifically exposed to neutron or mixed neutron/x-ray irradiation. We will gain further insight by focusing on the bone marrow mononuclear cells and hematopoietic stem cells, two cellular compartments that are the targets of RP gene defects that impair their function [46, 47].

## Conclusions

Our data provide evidence that biological processes related to mRNA translation are significantly underrepresented following neutron exposure, even at the smallest neutron percentage (5%). In addition, tRNA processes are also downregulated in the 83% neutron exposure. Among the differentially expressed genes, neutron exposure results in the downregulation of multiple ribosomal protein genes. In humans, loss of function of many *RP* genes and ribosome dysfunction has been associated with bone-marrow failure syndromes. In fact, many of the *RP* genes mutated in Diamond-Blackfan syndrome are the same as the *RP* genes downregulated after exposure to neutron irradiation. The observation that these genes are downregulated by even a 5% neutron component in a mixed-field radiation exposure attests to the established fact that neutrons are able to cause much more severe DNA damage compared to photons [54]. It is therefore important for emergency planners to be able to detect the neutron component in the case of a detonation of an IND.

## Additional files

Additional file 1: Excel file with 5 tabs containing a summary of differentially expressed genes by radiation type. (XLSX 2429 kb)

Additional file 2: Excel file with complete output of IPA canonical pathways. The “EIF2 Signaling” pathway and the related “mTOR Signaling”, “Regulation of eIF4 and p70S6K Signaling”, and “p70S6K Signaling” pathways are highlighted in red. The most statistically significant canonical pathways (Benjamini-corrected *p* value < 0.05) are listed according to the negative log of the p value. (XLSX 50 kb)

Additional file 3: **Figure S1.** Ingenuity Pathway Analysis (IPA) on EIF2-related differentially expressed genes by applying a fold-change cutoff 2 vs. 1.5 plotted according their *p*-value (−log). Dotted vertical line corresponds to a p value of 0.05. (PDF 19 kb)

Additional file 4: Excel file with complete output of PANTHER biological processes. Benjamini-corrected *p* values are shown. (XLSX 32 kb)

Additional file 5: List of differentially expressed genes included in the “EIF2 signaling” canonical pathway identified by IPA core analysis. Differentially expressed genes specific to neutron radiation are depicted in *red*. Genes unique to x-ray exposure are depicted in *green*. (XLSX 14 kb)

Additional file 6: **Table S1.** Protein ubiquitination processes identified by PANTHER analysis. Benjamini-corrected p values are shown. (PDF 30 kb)

Additional file 7: IPA-predicted upstream regulators of gene expression. Benjamini-corrected p values are shown. Top predicted upstream regulators (|z| > 2) are shown. (XLSX 11 kb)

Additional file 8: **Figure S2.** Comparison of fold-change of *Crip2*, *Chst3*, *Add3*, *Eif3f*, *Rpl26*, *Rpl27*, *Rps17*, *Rps19*, and c*-Myc* by qPCR and DNA microarray. (PDF 61 kb)

## Abbreviations

DBA: Diamond-Blackfan Anemia
FDR: False Discovery Rate
IND: Improvised Nuclear Device
IPA: Ingenuity Pathway Analysis
PANTHER: Protein ANalysis THrough Evolutionary Relationships
qRT-PCR: quantitative Real-Time Polymerase Chain Reaction
RBE: Relative Biological Effectiveness
RP: Ribosomal Protein
