# Supplementary figures and images for: Identification of differentially expressed genes and pathways in mice exposed to mixed field neutron/photon radiation
Source: BMC Genomics. 2018 Jun 28;19:504. doi: 10.1186/s12864-018-4884-6 (PMC6027792; doi:10.1186/s12864-018-4884-6)

**Figure S1**

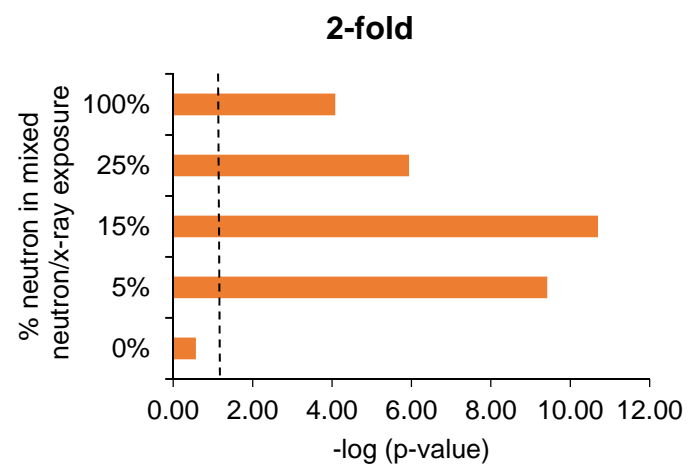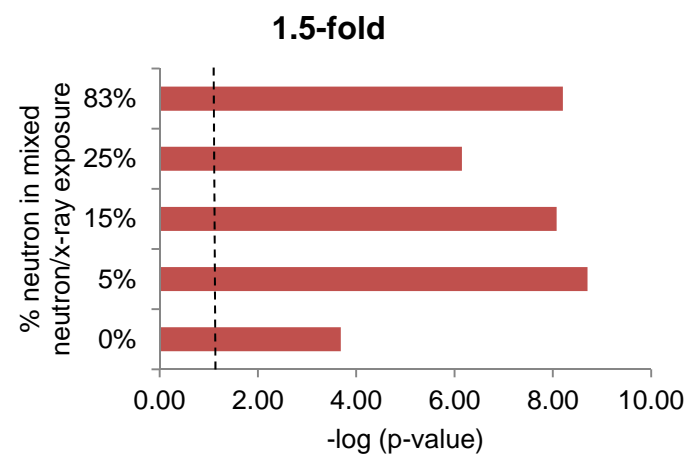

Supplement: Supplementary file 3 — Figure S1. Ingenuity Pathway Analysis (IPA) on EIF2-related differentially expressed genes by applying a fold-change cutoff 2 vs. 1.5 plotted according their p-value (−log). Dotted vertical line corresponds to a p value of 0.05. (PDF 19 kb) [file 12864_2018_4884_MOESM3_ESM.pdf]

Figure S2

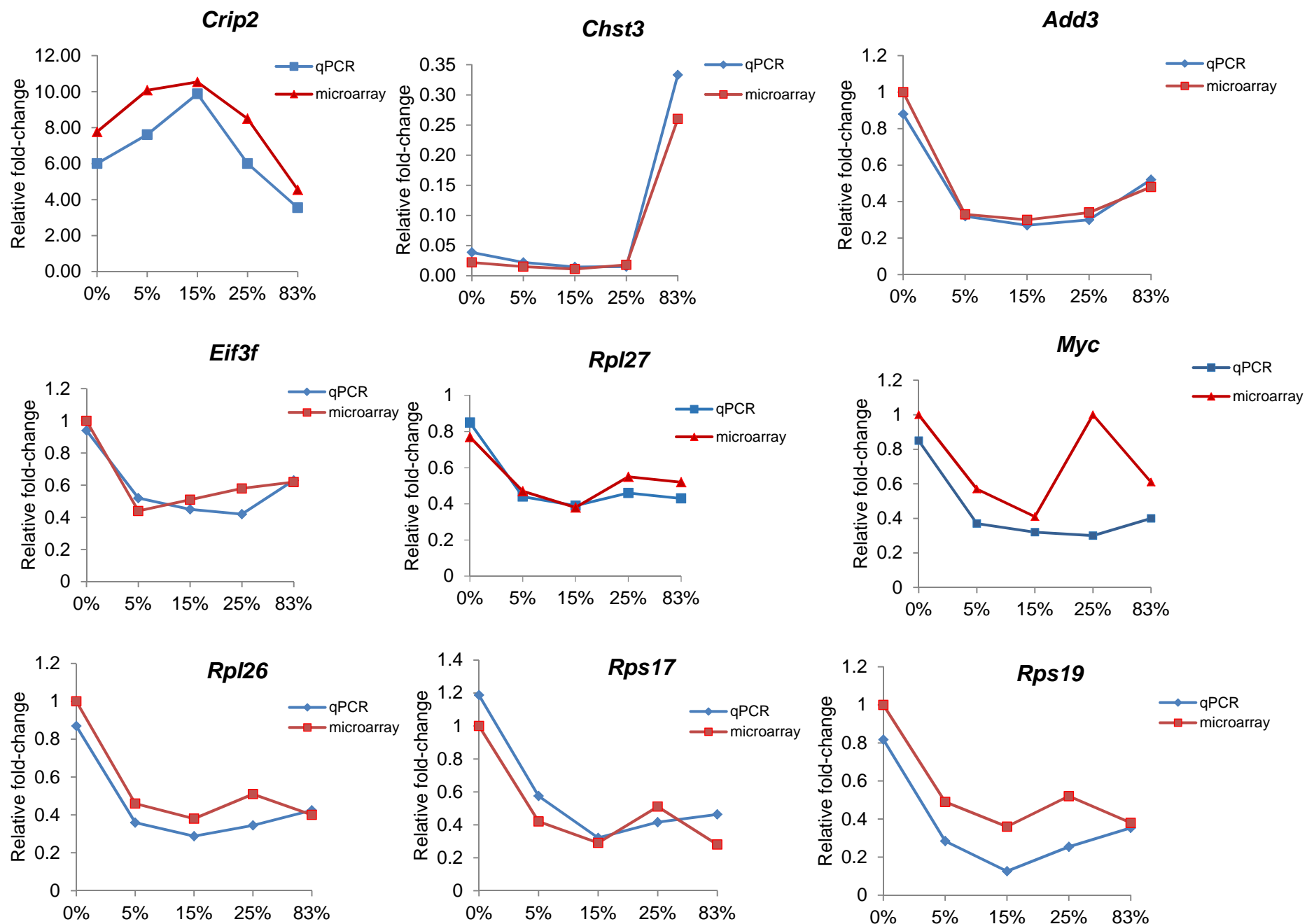

Supplement: Supplementary file 8 — Figure S2. Comparison of fold-change of Crip2, Chst3, Add3, Eif3f, Rpl26, Rpl27, Rps17, Rps19, and c-Myc by qPCR and DNA microarray. (PDF 61 kb) [file 12864_2018_4884_MOESM8_ESM.pdf]
