# Supplementary material for: Identification of differentially expressed genes and pathways in mice exposed to mixed field neutron/photon radiation
Source: BMC Genomics. 2018 Jun 28;19:504. doi: 10.1186/s12864-018-4884-6 (PMC6027792; doi:10.1186/s12864-018-4884-6)
Supplement: Supplementary file 6 — Table S1. Protein ubiquitination processes identified by PANTHER analysis. Benjamini-corrected p values are shown. (PDF 30 kb) [file 12864_2018_4884_MOESM6_ESM.pdf]

Table S1

Protein ubiquitination pathways upregulated in response to radiation exposure as identified by PANTHER analysis (p<0.005).

Term	0%	5%	15%	25%	83%
ubiquitin protein ligase activity (GO:0061630)	4.67E-10	7.01E-06	2.55E-08	3.73E-07	1.58E-09
ubiquitin-like protein ligase activity (GO:0061659)	7.67E-10	9.53E-06	3.99E-08	5.41E-07	2.55E-09
ubiquitin-like protein transferase activity (GO:0019787)	2.11E-09	7.75E-04	4.67E-06	1.76E-05	3.74E-10
ubiquitin-protein transferase activity (GO:0004842)	7.57E-09		6.63E-06	3.05E-05	1.37E-09
ubiquitin protein ligase binding (GO:0031625)	9.33E-08	6.78E-06	6.62E-06	5.15E-06	3.50E-06
ubiquitin-like protein ligase binding (GO:0044389)	2.84E-07	2.58E-06	6.66E-07	8.97E-07	1.73E-06
ubiquitin binding (GO:0043130)	7.91E-07	1.92E-05	1.86E-06	1.43E-05	7.55E-04
ubiquitin-like protein binding (GO:0032182)	1.45E-06	6.36E-05	1.32E-05	1.54E-05	7.44E-04
polyubiquitin modification-dependent protein binding (GO:0031593)	1.79E-06	6.54E-07	1.75E-05	2.23E-06	
ubiquitin conjugating enzyme activity (GO:0061631)	4.94E-06		2.53E-04	1.64E-03	5.05E-06
ubiquitin-like protein conjugating enzyme activity (GO:0061650)	6.58E-06		3.16E-04	1.95E-03	6.72E-06
thiol-dependent ubiquitinyl hydrolase activity (GO:0036459)	2.21E-04	1.84E-03			
ubiquitinyl hydrolase activity (GO:0101005)	2.21E-04	1.84E-03		1.16E-02	1.21E-02
ubiquitin-like protein-specific protease activity (GO:0019783)			8.88E-03	1.09E-02	2.73E-02
ubiquitin-like protein conjugating enzyme binding (GO:0044390)	4.70E-03		2.11E-02		1.30E-03
deubiquitinase activator activity (GO:0035800)	6.49E-03				
ubiquitin conjugating enzyme binding (GO:0031624)	7.28E-03		3.19E-02		
ubiquitin-specific protease binding (GO:1990381)	1.59E-02	3.95E-02	3.81E-03	1.03E-02	
K63-linked polyubiquitin modification-dependent protein binding (GO:0070530)	4.34E-02	2.03E-02		2.90E-02	
K48-linked polyubiquitin modification-dependent protein binding (GO:0036435)		4.49E-03	1.04E-02	6.11E-03	
ubiquinol-cytochrome-c reductase activity (GO:0008121)		4.49E-03		6.11E-03	
ubiquitin-like protein-specific protease activity (GO:0019783)		4.74E-03			
ubiquitin-specific protease activity involved in negative regulation of ERAD pathway (GO:1904455)		4.54E-02			
ubiquitin-specific protease activity involved in negative regulation of retrograde protein transport, ER to cytosol (GO:1904265)		4.54E-02			
ubiquitin modification-dependent histone binding (GO:0061649)			1.58E-02		
ubiquitin protein ligase activity involved in ERAD pathway (GO:1904264)			2.63E-02		
ubiquitin activating enzyme activity (GO:0004839)			2.70E-02		
ubiquitin conjugating enzyme binding (GO:0031624)					4.59E-04
ubiquitin-ubiquitin ligase activity (GO:0034450)					2.87E-02

Significantly enriched biological processes among genes upregulated in mouse blood after x-ray, neutron, or mixed field x-ray/neutron treatment relative to unirradiated controls ($p < 0.005$). Benjamini-corrected p values are shown.
